# The budget impact of patterned frequency-modulated oral stimulation to promote non-nutritive sucking in preterm infants in the United States

**DOI:** 10.3389/fped.2025.1688146

**Published:** 2025-12-01

**Authors:** Tobias Muench, Carla Fernández Barceló, Alex Veloz, Rhodri Saunders

**Affiliations:** 1 Coreva Scientific, Koenigswinter, Germany; 2 HEOR Pro, Geneva, IL, United States

**Keywords:** economic evaluation, premature birth, non-nutritive sucking, NICU, gavage feeding, nasogastric tube

## Abstract

**Background:**

Premature childbirth interrupts in-utero development of essential functionalities including non-nutritive sucking (NNS), a pre-requisite for full oral feeding (FOF), often a requirement for hospital discharge. Patterned and frequency-modulated oro-somatosensory stimulation (PFOS) can help preterm infants develop NNS. This study assessed the health-economic impact of PFOS in preterm infants in the United States (US).

**Methods:**

A budget impact analysis modeled the hospital care pathway for preterm infants through a decision tree and Markov model with a 5-year time horizon. The analysis examined 100 hypothetical preterm infants with gestational age at birth (GAB) 25–30 weeks, using PFOS (NTrainer™) or standard of care (SoC). Input data were taken from published literature. Key outcomes were total costs, neonatal intensive care unit (NICU) days, infections, and rehospitalizations. Probabilistic and one-way sensitivity analyses were performed to address uncertainty.

**Results:**

For 100 preterm infants, insurance payer costs were estimated at $21,713,932 and $24,063,242 for PFOS and SoC, respectively. On average, costs with PFOS were lower by $2,349,309 (95% uncertainty interval (UI): −$342,130; $4,945,657). Sensitivity analysis showed that PFOS was cost saving in 96% of simulations. Results were driven by reduced time to FOF and discharge (−577 NICU days), while decreasing infections and rehospitalizations. From the hospital perspective, the model resulted in total costs of $8,746,157 with PFOS and $9,736,209 with SoC, a difference of $990,051 (95% UI: -$163,528; $2,093,355). Introducing PFOS was cost saving for hospitals in 96% of the simulations according to the model estimates.

**Conclusion:**

PFOS is expected to reduce the cost of care associated with developing NNS in preterm infants in the US from both a payer and hospital perspective, being especially cost-effective in older preterm infants (GAB 29–30).

## 1 Introduction

Having a functional feeding capacity is a critical developmental milestone for newborns, facilitating nutrition intake and establishing the foundation for long-term growth and health. In full-term infants, oral feeding competency occurs as a coordinated interplay of sucking, swallowing, and breathing that develops progressively throughout gestation (1). However, in preterm infants, these feeding capabilities are frequently underdeveloped due to developmental interruptions following premature birth. Frequently, they are further compromised by medical interventions necessary for survival, such as intubation, positive airway pressure therapy, and the application of adhesive devices around the oral area (2, 3).

Dysfunctional oral feeding is a major cause of morbidity in preterm infants and has been associated with prolonged need for parenteral nutrition, prolonged hospital stay, and complications such as sepsis, cholestasis, and malnutrition (4, 5). The impact on the newborn is not just short term, as evidenced by poor neurodevelopmental and digestive outcomes that persist into childhood and adulthood (4, 5). Parents are also often affected, with prolonged neonatal intensive care unit (NICU) stays and feeding challenges leading to significant stress and disruption to sleep, work, and overall well-being (6–9).

A key aspect of care for preterm newborns is helping them to develop their non-nutritive sucking (NNS), a pre-requisite for nutritive sucking and independent feeding (10, 11). Achieving full oral feeding (FOF) is a critical factor for hospital discharge of most newborns. Current standard-of-care interventions to promote FOF in preterm infants often include sensory stimulations such as tactile, kinetic, and auditory modalities, which can help develop NNS. Among these, oral stimulation has been extensively studied and is delivered using either a pacifier under caregiver supervision or oral-motor interventions administered by therapists and/or nurses (12). These interventions have generally demonstrated clinical benefits vs. no oral stimulation, such as reduced time to FOF and shorter NICU lengths of stay (13).

A new alternative is patterned frequency-modulated oro-somatosensory stimulation (PFOS), which uses a pneumatically pulsed pacifier to deliver controlled oral stimulation (14). Evidence from a randomized controlled trial suggests that PFOS improves feeding outcomes compared to non-pulsatile pacifiers (15). How the clinical efficacy translates into health-economic impact remains unexplored, representing a critical gap in the literature as well as a potential hurdle towards PFOS uptake in clinical practice. This study aims to address this gap by evaluating the budget impact of PFOS use in the NICU setting from an insurance payer and hospital perspective.

## 2 Materials and methods

### 2.1 Analytic approach

To estimate the budget impact of introducing PFOS into the care pathway for preterm newborns in the United States (US), a decision-analytic model was developed in Microsoft Excel™ (Redmond, US). The model evaluated the cost consequences of integrating PFOS as a NNS training intervention compared to the standard of care (SoC). Model results were inflated to and reported in 2024 US Dollars (USD, $). In accordance with the Professional Society for Health Economics and Outcomes Research (ISPOR) good practice guidelines for budget impact models, discounting was not applied in the model (16). The primary model outcome was the budget impact expressed in total costs, while parameters such as NICU days, discharges with nasogastric tube (NGT), infections in- and outside the hospital, and number of rehospitalizations were tracked for contextualization. This publication reports in accordance with the Consolidated Health Economic Evaluation Reporting Standards (CHEERS 2022) checklist (17).

### 2.2 Patient population

The patient population in the model reflected preterm newborns between 25- and 30-weeks gestational age at birth (GAB) in the US. The 100-patient cohort was constructed to have mean characteristics matched to those in the clinical trial published by Song et al. (15).

### 2.3 NNS training delivery

SoC for promoting NNS was taken as defined in by Song et al. (15), namely oral stimulation using pacifiers or fingers delivered by a nurse or by a caregiver. Patients in the intervention arm used PFOS for promoting development of NNS. For both SoC and PFOS, clinical data were taken from Song et al. (15), in which PFOS data were specific to the NTrainer™ system (Cardinal Health™, Dublin, Ohio). Both intervention and comparator training protocols are assumed to be in line with those applied in the clinical trial.

### 2.4 Model structure

**Figure 1:**
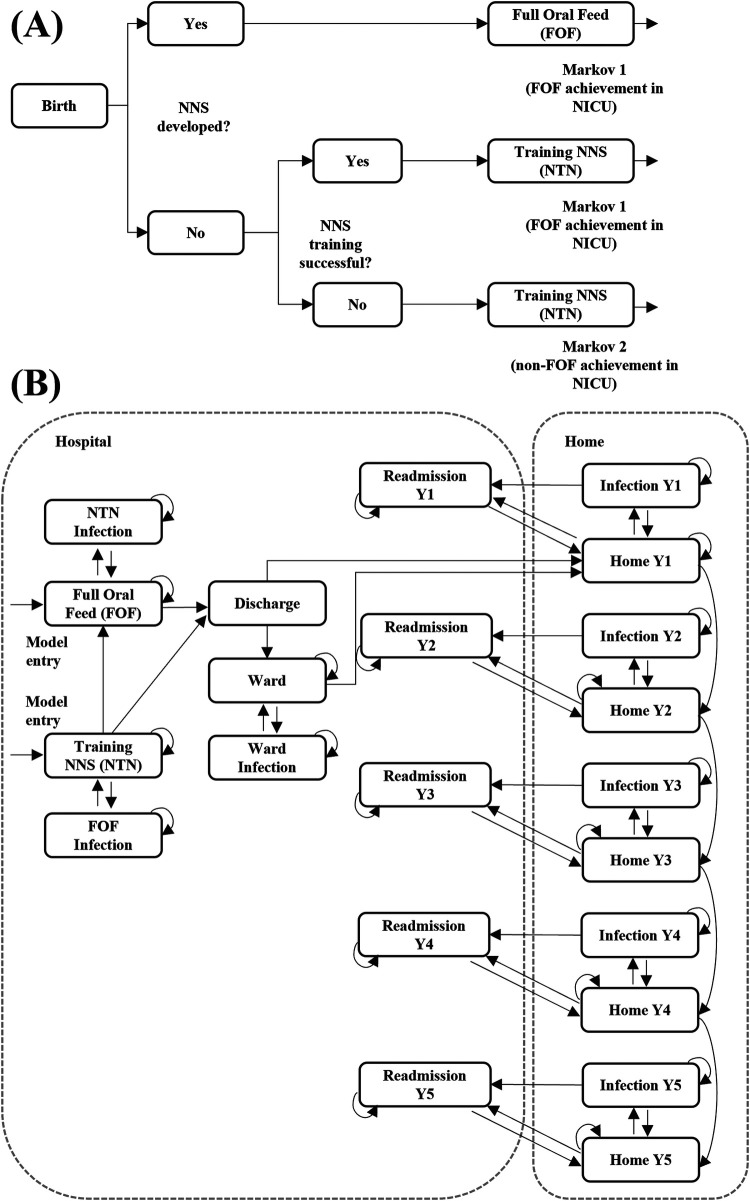
Model structure: **(A)** decision tree **(B)** Markov model. Full model structure over a time horizon of five years (payer perspective). NICU, neonatal intensive care unit; NNS, non-nutritive suck; Y, year.

The patient pathway was modeled as a decision tree plus a Markov model (Figure 1) with daily cycles up to 180 days and half-monthly cycles from 180 days onwards. The decision tree aimed to pre-stratify the cohort based on the (non-)achievement of FOF. These cycles are chosen to capture the in-hospital transitions as accurately as possible in the Markov structure while the rehospitalization and infection rates after discharge could be captured in a less granular way, and every 2 weeks was sufficient. Through this stratification, the derived proportions of the cohort entered different Markov models. A Markov model structure was chosen to represent the disease pathway since the patients could move between states at different proportions. The simplicity and transparency of the Markov model structure allowed us to easily incorporate clinical trial data. There were two Markov models, identical in structure but having different transition probabilities. In this way, the sub-cohort who achieved and did not achieve FOF could progress appropriately through the care pathway.

In the decision tree, patients entered the state of “Birth” as preterm newborns between 25- and 30-weeks GAB in the NICU. From there, patients with NNS already developed transitioned directly into the achieved-FOF state of Markov 1. Patients with NNS not developed at birth could either have successful or unsuccessful NNS training. Pre-stratification was undertaken by funneling the patients into two different Markov models based on predicted FOF achievement. Patients who would be trained to achieve FOF in NICU successfully, would enter Markov 1. Patients who would be trained but eventually not achieve FOF in the NICU would be stratified into Markov 2.

Entry states to the Markov model were in the NICU and were the achieved-FOF state of the NNS training state. Patients with FOF achievement (Markov 1) or without FOF achievement (Markov 2) could either be discharged from NICU to home or have an additional hospital stay in a lower acuity ward. However, the exact discharge behavior of both groups were not apparent in the clinical publication (15). Patients who did not achieve FOF were assumed to have a longer discharge pattern from the NICU. To account for this, a variable was introduced to modulate time to discharge (TTD). If TTD was 1 then the discharge transition probability was the same as with successful FOF. A TTD >1 resulted in a higher transition probability towards discharge, thus shortening the average NICU length of stay. A TTD <1 resulted in lower transition probability towards discharge, leading to longer NICU length of stay. In the base case TTD was set at 0.8 and was varied in sensitivity analyses. Patients in all hospital states were at risk of infection.

At home, patients could either remain in a healthy state, develop a home-managed infection, or develop an infection that required rehospitalization. Rehospitalizations were retrieved from a study that split rates by GAB 24–27 and GAB 28–31 (18). Weighted rehospitalization risks were calculated based on the proportions of patients in the mixed GAB 25–30 group. The follow-up period at home was captured for up to 5 years. At the end of each year, the patient moved to the next-year Markov state cluster. At any point in time patients could die either in hospital or at home.

Costs per member per month (PMPM) were calculated for the 100-patient cohort by retrospectively constructing the number of insurance payer lives covered. For this, the proportions of women of reproductive age, the annual fertility rate in the US, and the proportion of births being prematurely born between GAB 25–30 were incorporated.

#### 2.4.1 Insurance payer perspective

Analyses from the insurance payer perspective used the full-time horizon of the model (5 years). Costs in each state of the Markov model were based on hospital charges per day and on costs per event for infections and readmissions in the home setting.

#### 2.4.2 Hospital perspective

Analyses from the hospital's perspective used only the first year of the model time horizon. Costs for infections at home were not included. Hospital-level costs and staff-time costs for NNS training were included.

### 2.5 Model input data

A structured literature review in PubMed was conducted in April 2024 to retrieve peer-reviewed, published data to populate the model. The literature search strategy can be found in Supplementary Table S1. Inputs that were not retrieved from the structured literature search, were extracted through targeted searches and grey literature. Where peer-reviewed published data were not available to inform of a required input variable, conservative assumptions were used and validated via sensitivity analyses. Clinical data were primarily based on the multi-center clinical trial from Song et al. (15). Monetary values are presented in 2024 USD, with hospital charges used for the payer perspective and hospital costs for the hospital perspective. Whenever only costs or charges were available, a cost-to-charge ratio was applied to estimate the input. Where required, costs were inflated to 2024 USD. The input data can be found in Supplementary Table S2.

### 2.6 Model uncertainty

Uncertainty was assessed via one-way and probabilistic analyses. The one-way sensitivity analysis was constructed to estimate the impact of single parameters on the results, varying each parameter by ±20% of the respective base case input. The 10 parameters with the highest impact on the results were identified and plotted in a tornado diagram. In a univariate sensitivity analysis, the TTD parameter (0.8) assumption was varied stepwise increasing the factor by 0.1 from 0.5 to 1.2.

Parameter uncertainty was assessed in the probabilistic analysis via a 5,000-iteration Monte Carlo simulation. Each parameter was assigned a distribution, either using literature data or uncertainty assumptions (10% for general parameters, 20% for costs). Normal distributions were applied to continuous variables and mid-range proportions (10%–90%), while beta distributions were used for proportions near the extremes (<10% or >90%) to account for boundary constraints. Log-normal and Gamma distributions were used for odds ratios and costs/charges, respectively. During the Monte Carlo simulation, each input of the model was varied. The methods to conduct the Monte Carlo simulation were described elsewhere (19). Results were captured as the 95% uncertainty interval (UI) characterizing the lower and upper bounds of 95% of the Monte Carlo simulations.

A threshold analysis was conducted to assess the impact of PFOS's costs on the total savings. An arbitrary cost of $5,000 per patient was assumed for the base case. Increments and decrements of the cost of PFOS (from $1,000 to $20,000) were plotted against the calculated cost savings based on the 5,000-iteration Monte Carlo simulation.

### 2.7 Model validation

All model components, including structure, logic, and assumptions, were reviewed by experts in health economics to ensure clinical relevance and methodological soundness. Internal model validation included formula audits, logic checks, and stress testing of transition probabilities and cost calculations. The model was developed in accordance with the Consolidated Health Economic Evaluation Reporting Standards (CHEERS 2022) and the ISPOR Good Practices for Budget Impact Analysis, ensuring transparency, reproducibility, and alignment with international standards. Extensive one-way and probabilistic sensitivity analyses were conducted to assess the impact of parameter uncertainty and model assumptions on outcomes.

## 3 Results

### 3.1 Model inputs

The input data for the model, derived from the structured literature review and hand searches, are presented in Supplementary Table S2. Inflation indices can be found in Supplementary Table S3.

### 3.2 Payer model

From an economic perspective, the model resulted in a cost saving of $2,349,309 USD (95% Uncertainty Interval, UI: −$342,130; $4,945,657) for a cohort of 100 preterm newborns when using PFOS compared to the SoC over a time horizon of 5 years. The total costs per arm were $21,713,932 and $24,063,242 for the PFOS and the SoC, respectively. Across both arms, roughly 91.9% of the total costs were attributable to NICU stay level III, followed by 7.4% of costs attributable to rehospitalizations. The rest of the costs were attributable to the lower acuity ward stay (NICU level I), infections in the hospital and at home, and discharges with NGT. The cost savings were 99% driven by the difference in NICU costs (Table 1). When breaking down the costs by FOF and non-FOF groups, the majority of the cost savings were attributable to the FOF group, mostly in the “NNS training and follow up” stage (Supplementary Table S4). The probabilistic sensitivity analysis showcased that PFOS treatment resulted in cost savings in 96% of the 5,000 Monte Carlo simulations, while the 95% uncertainty interval ranged from additional costs of up to $342,130 (lower bound) to cost savings up to $4,945,657 (upper bound).

**Table 1:** Economic impact by care setting for the insurance payer model.

	PFOS	Standard of care	Difference (95% UI)
In hospital			
NICU	$19,867,218	$22,182,414	−$2,315,196 (−$4,915,404; $383,574)
Lower acuity ward	$89,129	$97,219	−$8,090 (−$38,719; $20,676)
Infection	$15,101	$17,541	−$2,440 (−$5,423; $462)
At home			
Discharged with NGT	$24,696	$22,885	$1,811 (−$3,242; $7,058)
Infection	$43,995	$45,078	−$1,083 (−$1,839; −$424)*
Rehospitalizations	$1,673,794	$1,698,105	−$24,311 (−$49,082; −$4,507)*
Total costs	**$21,713,932**	**$24,063,242**	**−$2,349,309 (−$4,945,657; $342,130)**

PFOS, patterned frequency-modulated oral stimulation; NICU, neonatal intensive care unit; FOF, full oral feed; NGT, naso-/oro-gastric tube; UI, uncertainty interval.

* Statistically significant (*p* < 0.05).

Costs reported as 2024 US dollars. Values in bold are column totals.

From an insurance payer perspective, for a total of 982,791 lives covered, the results of 100 patients translated into costs per member per month of $0.37 per PFOS and $0.41 per SoC arm, saving $0.04 (95% UI: −$0.01; $0.08) per member per month.

When investigating the clinical and resource results of the model, the results were driven by 577 (95% UI: −109; 1,260) saved NICU days (Table 2). Use of PFOS resulted in reduced in-hospital infections (1.10 per 100 newborns, 95% UI −0.21; 2.44) and lower home infections (0.88 per 100 newborns, 95% UI 0.66; 1.25). The use of PFOS resulted in an increase in the number of discharges with naso-gastric tubes by 0.48 (95% UI −0.83; 1.85) per 100 patients. There was also a reduction in rehospitalizations with use of PFOS, with 1.97 (95% UI 1.19; 3.06) rehospitalizations prevented per 100 preterm babies over a time horizon of 5 years.

**Table 2:** Clinical results for the insurance payer model for 100 patients.

	PFOS	Standard of care	Difference (95% UI)
In hospital			
NICU days (in days)	4,852	5,429	−577 (−1,260; 109)
Number of infections due to NGT	6.65	7.75	−1.10 (−2.44; 0.21)
At home			
Discharged with NGT (number of patients)	6.42	5.94	0.48 (−0.83; 1.85)
Number of infections	42.11	42.99	−0.88 −1.25; −0.66)*
Number of rehospitalizations	121.01	122.98	−1.97 (−3.06; −1.19)*

PFOS, patterned frequency-modulated oral stimulation; NICU, neonatal intensive care unit; NGT, naso-/oro-gastric tube; UI, uncertainty interval.

* Statistically significant (*p* < 0.05).

A threshold analysis based on the 5,000 Monte Carlo simulation revealed that the PFOS device would be cost saving in 91.1% of cases from a payer perspective if the capital costs of the device, implementation costs and operational costs were below an arbitrary total cost of $5,000 per patient. This would be roughly 1.5 NICU days, for contextualization.

### 3.3 Hospital model

From a hospital perspective, over a time horizon of one year, using PFOS led to savings of $990,051 (95% UI: −$142,899; $2,084,724). Similar to the insurance payer model, the majority of the costs were attributable to NNS training and follow up in the FOF patient group (Table 3). Freed up staff training time by using PFOS, which was included in the hospital adaptation of the model, created cost savings of −$257 in the non-FOF group and −$12,297 in the FOF group. Examining the results of the 5,000-iteration Monte Carlo analysis, it could be observed that PFOS led to cost savings in 96% of the iterations.

**Table 3:** Economic impact results by FOF achievement for the hospital model.

	PFOS	Standard of care	Difference (95% UI)
FOF group			
Pre NNS training NICU	$3,092,177	$3,331,773	−$239,596 (−$879,858; $397,517)
NNS training and follow up	$4,938,498	$5,652,845	−$714,347 (−$1,516,179; $76,766)
Staff time for training	$66,431	$78,728	−$12,297 (−$26,825; $1,776)
Non-FOF group			
Pre NNS training NICU	$212,208	$210,428	$1,780 (−$59,727; $60,029)
NNS training and follow up	$430,194	$455,529	−$25,335 (−$136,982; $87,438)
Staff time for training	$6,649	$6,906	−$257 (−$2,009; $1,497)
Total costs	**$8,746,157**	**$9,736,209**	**−$990,051 (−$2,084,724; $142,899)**

PFOS, patterned frequency-modulated oral stimulation; FOF, full oral feed; NNS, non-nutritive sucking; NICU, neonatal intensive care unit; UI, uncertainty interval.

Costs reported as 2024 US dollars. Values in bold are column totals.

### 3.4 One-way sensitivity analysis

**Figure 2:**
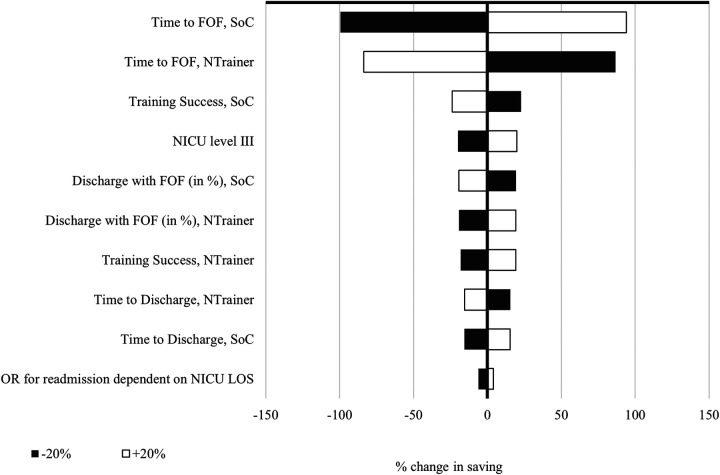
One-way sensitivity analysis for the payer model. NTrainer, patterned frequency-modulated oral stimulation (PFOS) using the NTrainer device; SoC, standard of care; FOF, full oral feed; NNS, non-nutritive sucking; NICU, neonatal intensive care unit; LOS, length of stay; OR, odds ratio.

**Figure 3:**
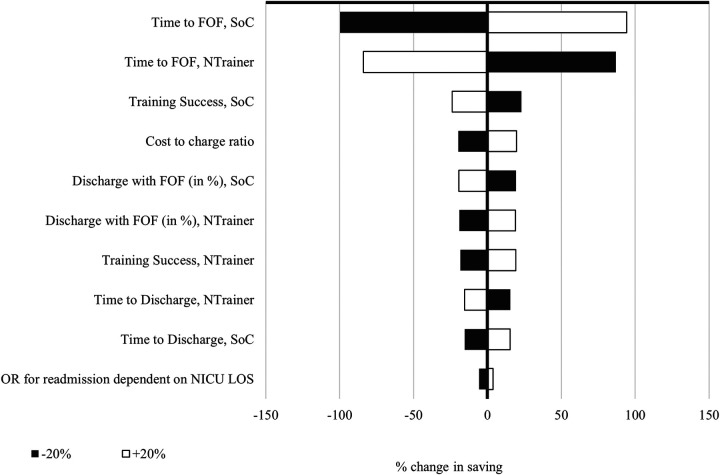
One-way sensitivity analysis for the hospital model. NTrainer, patterned frequency-modulated oral stimulation (PFOS) using the NTrainer device; SoC, standard of care; FOF, full oral feed; NNS, non-nutritive sucking; NICU, neonatal intensive care unit; LOS, length of stay; OR, odds ratio.

The variables that most impacted model outcomes for payers and hospitals can be seen in Figures 2, 3. While the individual percentage influence of the single parameter differed slightly across the models, the relative impact was the same. Time to achieve FOF was the most impactful variable as could be expected given that it largely determines time in the NICU, which accounts for the vast majority of costs incurred. The only parameter that drove the analysis results for the hospital model and was not present in the payer model was the applied charge-to-cost ratio, since most of the NICU costs used in the model were transformed from charges to hospital costs. Notably, the TTD factor of 0.8 did not have a major influence on the results.

In the univariate sensitivity analysis, the TTD factor was varied from 0.5 to 1.2. The maximum variation in the cost difference resulted from keeping the TTD parameter at 1.0 (0.41% or $9,555). The results using the default of 0.8 created a total variation in the cost difference of $6,747, which translates into a small relative difference of 0.29% (see Supplementary Table S5).

The threshold analysis to assess the impact of PFOS's costs per patient showed that the probability of PFOS being cost saving ranked above 90% when device costs were up to $5,000. The graphical representation of the threshold analysis can be seen in Supplementary Table S1.

### 3.5 Subgroup analysis

The subgroup analysis, stratified by GAB groups based on the clinical trial, revealed higher cost savings for the older age group (Table 4). This is in line with the trial data inputs, where the older age group achieved significantly shorter NICU stays comparing PFOS and the SoC. Overall, the cost savings decreased to $1,807,593 when inputting the sub-group specific data, where 89.7% of cost savings were attributed to the GAB 29–30 group. The probabilistic sensitivity analysis revealed that in the GAB 29–30 age group PFOS resulted in cost savings of $1,621,890 (95% UI $175,251; $3,081,563) with 99.5% of simulations being favorable for PFOS. In the GAB 25–28 age group, PFOS was cost saving in 62% of cases and resulted in savings of $185,704 (95% UI −$2,088,657; $2,439,539), showcasing high uncertainty and variability.

**Table 4:** Economic impact results by GAB for the hospital model.

	PFOS	Standard of care	Difference (95% UI)
GAB 25–28	$13,824,084	$14,009,788	−$185,704 (−$2,439,539; $2,088,657)
GAB 29–30	$8,092,140	$9,714,029	−$1,621,890 (−$3,081,563; −$174,251)
Total costs	**$21,916,224**	**$23,723,817**	**−$1,807,593 (−$4,374,042; $896,925)**

GAB, gestational age at birth; PFOS, patterned frequency-modulated oral stimulation; GAB, gestational age at birth; UI, uncertainty interval.

Costs reported as 2024 US dollars. Values in bold are column totals.

## 4 Discussion

Introducing PFOS for a cohort of 100 preterm newborns between 25 and 30 GAB was cost saving in 96% of the simulations in the payer model, with −$2,349,309 average cost savings. The model-based results were mainly driven by a shorter time to full-oral feed, leading to shorter NICU stays [−577 NICU days per 100 patients, in line with the clinical trial length of stay difference of 6 days per patient (15)]. In this model, there was a slight reduction in the infections due to naso-/orogastric tubes in the hospital and at home, as well as rehospitalizations. Nevertheless, the subgroup analysis revealed that PFOS is more cost saving for preterm newborns GAB 29–30 compared to GAB 25–28 ($1,621,890 and $185,704, respectively). The hospital model yielded similar results, with 96% of the simulations being cost saving ($990,051) when reducing the time horizon from 5 years to 1 year, and when not considering the home care related costs due to infections and rehospitalizations.

The model shows a slight non-statistically significant increase in discharges with naso-gastric tubes in the PFOS group (+0.48 per 100 patients), which seems counterintuitive given PFOS's role in accelerating full oral feeding (FOF). This finding could reflect an effect on care practices rather than a direct clinical drawback of PFOS. A plausible explanation could be that PFOS may accelerate readiness for discharge in infants who are otherwise stable but have not yet achieved FOF. In these cases, infants could be ready for discharge based on the existing hospital protocols. Clinicians might decide to discharge preterm newborns with naso-gastric tubes for home feeding, presuming preterm newborns trained with PFOS will transition to FOF faster at home. Another possible explanation could be that hospitals using PFOS may adopt early discharge protocols, prioritizing reduced NICU stay over FOF achievement, especially if PFOS is promising in continued feeding development post-discharge.

Downstream implications of a higher rate of naso-gastric tube discharge could be that home care costs increase slightly due to the need for naso-gastric tube management, supplies, and potential home nursing support. Rehospitalization risk could rise if naso-gastric tube complications occur, although the model shows a net reduction in rehospitalizations with PFOS. In terms of quality of life, parental burden may increase due to the need to manage naso-gastric tubes at home, potentially affecting sleep, stress, and caregiving experience. However, earlier discharge may also improve family bonding, reduce hospital-related stress, and allow for more natural developmental environments.

This work did not take the product pricing of the PFOS solution into account, which will impose additional costs on the hospital. Following our threshold analysis, using PFOS at an arbitrary product cost and running cost of $5,000 could yield cost savings in 91.1% of the simulations from a payer perspective.

The model-based results from implementing PFOS align with economic evidence from published studies. NICU stays are a primary cost driver, as NICU costs inversely scale with gestational age, with average costs increasing rapidly the more premature the infant is (20, 21). This is largely explained due to a prolonged NICU stay, higher complication rates and higher resource intensity for younger infants. The observed reduction in feeding tube-related infections and rehospitalizations in the model is also consistent with literature, where preterm newborns between 25 and 28 GAB had a 20.4% infection rate, similar to the 22.9% reported in the model over a 1-year time horizon (22). Preterm infants fed through naso-/orogastric tubes are at higher risk of late-onset sepsis due to the potential for bacterial colonization and biofilm formation on feeding tubes (23, 22). Late-onset sepsis is a major cause of morbidity and mortality in NICUs, with higher risk of developing sepsis when the time to FOF is longer (24). Faster achievement of FOF could reduce exposure to these infection-prone feeding methods, thereby lowering the incidence of infection. Moreover, the average length of stay used as model inputs (65.8 days for SoC, 59.8 for PFOS) are similar to the results of a large cohort study including over 20,000 preterm newborns (73.2 days for GAB 25–30) (25).

According to the clinical study by Song et al., the benefits of PFOS are most pronounced in infants ≥29 weeks gestation (15). This suggests that the budget impact for preterm cohorts spanning 25–30 weeks may vary depending on gestational age distribution, with greater savings anticipated in populations closer to 30 weeks. The economic implications were confirmed by the subgroup analysis, where the impact stratified by GAB 25–28 and GAB 29–30 was examined. Overall, it could be observed that the majority of savings (89.7%) were linked to the older age group. Reducing NICU stays by 4–10 days per infant aligns with established cost-effectiveness thresholds for neonatal interventions, which range from $1,000 to $9,100 per quality-adjusted life year (QALY) gained (26). This positions PFOS as a high-value intervention for moderate-to-late preterm infants, where shorter hospitalization drives substantial cost reductions.

When considering the long-term impact of faster FOF achievement, preterm infants who fail to achieve FOF by 40 weeks postmenstrual age face a 37% increased risk of adverse neurodevelopmental outcomes, including lower cognitive, language, and motor scores at 18–26 months (27). Moreover, preterm infants with feeding difficulties are 40% more likely to be diagnosed with a developmental coordination disorder at 4–5 years of age, compared to infants without feeding issues (28). As our model focused on infections and no other complicating factors, such as non-optimal neurodevelopment, the potential impact on payers may be higher than estimated here.

### 4.1 Strengths, limitations and future research

To our knowledge, the present study is the first evaluation of the economic impact of PFOS that has been published. This provides data to inform decisions as to whether using PFOS could be a clinically and economically beneficial intervention. Additionally, this study provides results from an insurance payer and a hospital perspective, giving information depending on the context and the level where decision making is being made. The main inputs for the model were retrieved from a randomized controlled trial (RCT), providing high quality data for variables that impact the clinical and resource-use outcomes the most.

However, this study yields several limitations that must be considered when interpreting the work. The main limitation is that the cost of the PFOS system has not been included in the analysis due to the changing nature of pricing and contracts in different hospitals, and payer contexts. We provided a threshold analysis with an arbitrary capital and operating cost per patient, as well as how this impacted the likelihood of it being cost saving. The hospital model focuses on costs and not reimbursement, providing a budget impact model for the hospital costs but not for the revenue. Moreover, the hospital costs were often not available, and a charge-to-cost ratio was used to derive the hospital costs. This charge-to-cost ratio might vary from institution to institution and therefore the hospital results might not be generalizable to all healthcare centers. Even though the main model inputs are retrieved from a randomized controlled trial, the population size for the trial was 100 preterm newborns and it was a single study, and thus, larger trials and observational studies could provide insights into the generalizability of these data. Lastly, wards can differ between hospitals, and while some hospitals might discharge newborns from the NICU to a step-down NICU ward (same physical space but lower intensity of care), others might discharge them to the general ward. The lower intensity NICU and general ward were assumed to cost the same in the present model since care provision without having achieved FOF was assumed to be similar. As shown in this work, reducing care intensity can lower total costs of healthcare provision in preterm infants dramatically.

Further research focusing on real-world data collection of the use of PFOS could shed more light on the results in terms of clinical and economic resources used, as there currently is only one published RCT on the effect of PFOS in preterm newborns. Moreover, if any new technological advancements and innovations to promote NNS in preterm newborns become available, additional cost-effectiveness evaluations should be carried out.

In conclusion, patterned frequency-modulated oral stimulation is expected to be a cost-saving strategy for preterm newborns from 25 to 30 weeks for most hospitals and payers based on the model estimates. When evaluating the preterm newborn subgroups by GAB, PFOS appears to be more cost-effective in the GAB 29–30 than in the GAB 25–28. In general, savings stem from a shorter time to achieve full oral feeding, which translates into shorter NICU stays. Larger studies to assess the clinical and economic impact of PFOS in different centers and contexts are advised to ensure the results are relevant for a broader audience.

## Data Availability

The original contributions presented in the study are included in the article/Supplementary Material, further inquiries can be directed to the corresponding author.
