# Supplementary material for: The budget impact of patterned frequency-modulated oral stimulation to promote non-nutritive sucking in preterm infants in the United States
Source: Front Pediatr. 2025 Dec 1;13:1688146. doi: 10.3389/fped.2025.1688146 (PMC12702860; doi:10.3389/fped.2025.1688146)
Supplement: Supplementary file 1 [file Table1.docx]

Supplementary Material

**Economic impact of patterned frequency-modulated oral stimulation to promote non-nutritive sucking in preterm infants**

Supplementary Table 1. Structured searches in PubMed to identify relevant modelling data.

| **Index** | Aim | Search string | Hits |
| --- | --- | --- | --- |
| 1 | Studies on PFOS | innara[tiab] OR ntrainer[tiab] OR "patterned and frequency-modulated oro-somatosensory stimulation (PFOS)"[tiab] | 6 |
| 2 | Studies on NNS | "oral stimulation"[tiab] OR "orocutaneous stimulation"[tiab] OR "non-nutritive sucking"[tiab] OR "non-nutritive suck"[tiab] OR ((sucking[tiab] OR suck[tiab] OR "oral feed"[tiab] OR "oral feeding"[tiab] OR suckle[tiab]) AND (pacifier[tiab] OR pacifiers[tiab])) OR "finger sucking"[tiab] OR "sucking finger"[tiab] OR finger-sucking[tiab] OR "non nutritive sucking"[tiab] OR "non nutritive suck"[tiab] OR oral-motor[tiab] OR oro-motor[tiab] OR oro-somatosensory[tiab] OR oral-somatosensory[tiab] | 2,207 |
| 3 | Studies on premature infants | (preterm[tiab] OR pre-term[tiab] OR premature[tiab] OR prematurity[tiab] OR preemie[tiab]) AND (newborn[tiab] OR infant[tiab] OR baby[tiab] OR babies[tiab] OR newborns[tiab] OR infants[tiab] OR children[tiab] OR child[tiab] OR birth[tiab] OR neonatal[tiab] OR neonate[tiab] OR neonates[tiab]) | 141,896 |
| 4 | Studies on oral and enteral feeding | "nasogastric tube"[tiab] OR "nasogastric tubes"[tiab] OR infections[tiab] OR infection[tiab] OR "oral feeding"[tiab] OR "full oral feeding"[tiab] OR "oral eating"[tiab] OR gavage[tiab] OR "enteral feeding"[tiab] OR "enteral nutrition"[tiab] OR "gastric tube"[tiab] OR "gastric tubes"[tiab] OR "feeding tube"[tiab] OR "feeding tubes"[tiab] OR "tube feeding"[tiab] OR tube-feeding[tiab] OR "oral feeds"[tiab] OR nippling[tiab] OR "bottle feeding"[tiab] OR "breast feeding"[tiab] OR breastfeeding[tiab] OR nursing[tiab] OR PO[tiab] | 2,255,793 |
| 5 | Studies on NICU | NICU[tiab] OR "neonatal intensive care unit"[tiab] OR "newborn intensive care unit"[tiab] OR "neonatal intensive care units"[tiab] OR "newborn intensive care units"[tiab] OR "neonatal ICU"[tiab] OR "newborn ICU"[tiab] | 30,829 |
| 6 | Studies on outcomes of interest | long-term[tiab] OR "long term"[tiab] OR rehospitalization[tiab] OR rehospitalisation[tiab] OR Rehospitalization[tiab] OR Rehospitalizations[tiab] OR rehospitalizations[tiab] OR rehospitalisations[tiab] OR (USD[tiab] OR dollar[tiab] OR dollars[tiab] OR "costs and cost analysis"[mh] OR "Health Care Costs/economics"[mh] OR cost-benefit[tiab] OR cost-effectiveness[tiab] OR cost-effective[tiab] OR cost-utility[tiab] OR cost-utilities[tiab] OR cost-consequence[tiab] OR "net monetary benefit"[tiab] OR cost-minimization[tiab] OR cost-minimisation[tiab] OR budget-impact[tiab] OR ((cost[tiab] OR costs[tiab] OR price[tiab] OR pricing[tiab] OR expense[tiab] OR expenditure[tiab] OR money[tiab] OR costing[tiab] OR budget[tiab] OR revenue[tiab] OR currency[tiab] OR expenses[tiab] OR budgets[tiab] OR currencies[tiab] OR expenditures[tiab] OR prices[tiab] OR revenues[tiab]) AND (study[tiab] OR analysis[tiab]))) | 1,739,946 |
| 7 | Studies on the specified time window | 2014/01/01:2024/03/01[edat] AND 2014/01/01:2024/03/01[dp] | 13,082,810 |
| 8 | Excluded study types | "Clinical Trial, Veterinary"[pt] OR "Case Reports"[pt] OR Address[pt] OR Autobiography[pt] OR "Clinical Conference"[pt] OR Biography[pt] OR Bibliography[pt] OR "Collected Work"[pt] OR Comment[pt] OR Congress[pt] OR "Historical article"[pt] OR Editorial[pt] OR "Expression of Concern"[pt] OR Dictionary[pt] OR Directory[pt] OR Interview[pt] OR "Interactive tutorial"[pt] OR "Legal Case"[pt] OR Lecture[pt] OR "Introductory Journal Article"[pt] OR Festschrift[pt] OR "Duplicate Publication"[pt] OR Legislation[pt] OR Letter[pt] OR News[pt] OR "Newspaper Article"[pt] OR "Observational Study, Veterinary"[pt] OR "Periodical Index"[pt] OR "Personal Narrative"[pt] OR Portrait[pt] OR "Patient Education Handout"[pt] OR "Published Erratum"[pt] OR "Retracted Publication"[pt] OR "Randomized Controlled Trial, Veterinary"[pt] OR "Retraction of Publication"[pt] OR "Twin Study"[pt] OR "Video-Audio Media"[pt] OR Webcast[pt] OR "Systematic Review"[pt] OR Review[pt] OR "Technical Report"[pt] | 8,581,979 |
| 9 | Excluded animal studies | See Supplementary text below on PubMed search string for animal studies | 7,952,967 |
| 10 | All exclusions | #8 OR #9 | 15,329,205 |
| 11 | All relevant studies on NTrainer™ | #1 | 6 |
| 12 | All relevant studies on NNS | #2 AND #3 AND #7 NOT #10 | 145 |
| 13 | All relevant studies on long-term effects of NICU stays | #4 AND #5 AND #6 AND #7 NOT #10 | 378 |

PFOS: Patterned Frequency-Modulated Oral Stimulation; NNS: Non-Nutritive Sucking; NICU: Neonatal Intensive Care Unit.

**PubMed search string for animal studies.**

Animal studies were identified using the search strings for PubMed and EMBASE as described by Hooijmans et al. 2010 (1).

"animal experimentation"[mh] OR "models, animal"[mh] OR invertebrates[mh] OR Animals[mh:NoExp] OR "animal population groups"[mh] OR chordata[mh:NoExp] OR "chordata, nonvertebrate"[mh] OR vertebrates[mh:NoExp] OR amphibians[mh] OR birds[mh] OR fishes[mh] OR reptiles[mh] OR mammals[mh:NoExp] OR primates[mh:NoExp] OR artiodactyla[mh] OR carnivora[mh] OR cetacea[mh] OR chiroptera[mh] OR elephants[mh] OR hyraxes[mh] OR Eulipotyphla[mh] OR lagomorpha[mh] OR marsupialia[mh] OR monotremata[mh] OR perissodactyla[mh] OR rodentia[mh] OR scandentia[mh] OR sirenia[mh] OR xenarthra[mh] OR haplorhini[mh:NoExp] OR strepsirhini[mh] OR platyrrhini[mh] OR tarsii[mh] OR catarrhini[mh:NoExp] OR cercopithecidae[mh] OR hylobatidae[mh] OR hominidae[mh:NoExp] OR "gorilla gorilla"[mh] OR "pan paniscus"[mh] OR "pan troglodytes"[mh] OR "pongo pygmaeus"[mh] OR ((animals[tiab] OR animal[tiab] OR mice[tiab] OR mus[tiab] OR mouse[tiab] OR murine[tiab] OR woodmouse[tiab] OR rats[tiab] OR rat[tiab] OR murinae[tiab] OR muridae[tiab] OR cottonrat[tiab] OR cottonrats[tiab] OR hamster[tiab] OR hamsters[tiab] OR cricetinae[tiab] OR rodentia[tiab] OR rodent[tiab] OR rodents[tiab] OR pigs[tiab] OR pig[tiab] OR swine[tiab] OR swines[tiab] OR piglets[tiab] OR piglet[tiab] OR boar[tiab] OR boars[tiab] OR "sus scrofa"[tiab] OR ferrets[tiab] OR ferret[tiab] OR polecat[tiab] OR polecats[tiab] OR "mustela putorius"[tiab] OR "guinea pigs"[tiab] OR "guinea pig"[tiab] OR cavia[tiab] OR callithrix[tiab] OR marmoset[tiab] OR marmosets[tiab] OR cebuella[tiab] OR hapale[tiab] OR octodon[tiab] OR chinchilla[tiab] OR chinchillas[tiab] OR gerbillinae[tiab] OR gerbil[tiab] OR gerbils[tiab] OR jird[tiab] OR jirds[tiab] OR merione[tiab] OR meriones[tiab] OR rabbits[tiab] OR rabbit[tiab] OR hares[tiab] OR hare[tiab] OR diptera[tiab] OR flies[tiab] OR fly[tiab] OR dipteral[tiab] OR drosphila[tiab] OR drosophilidae[tiab] OR cats[tiab] OR cat[tiab] OR carus[tiab] OR felis[tiab] OR nematoda[tiab] OR nematode[tiab] OR nematodes[tiab] OR sipunculida[tiab] OR dogs[tiab] OR dog[tiab] OR canine[tiab] OR canines[tiab] OR canis[tiab] OR sheep[tiab] OR sheeps[tiab] OR mouflon[tiab] OR mouflons[tiab] OR ovis[tiab] OR goats[tiab] OR goat[tiab] OR capra[tiab] OR capras[tiab] OR rupicapra[tiab] OR chamois[tiab] OR haplorhini[tiab] OR monkey[tiab] OR monkeys[tiab] OR anthropoidea[tiab] OR anthropoids[tiab] OR saguinus[tiab] OR tamarin[tiab] OR tamarins[tiab] OR leontopithecus[tiab] OR hominidae[tiab] OR ape[tiab] OR apes[tiab] OR pan[tiab] OR paniscus[tiab] OR "pan paniscus"[tiab] OR bonobo[tiab] OR bonobos[tiab] OR troglodytes[tiab] OR "pan troglodytes"[tiab] OR gibbon[tiab] OR gibbons[tiab] OR siamang[tiab] OR siamangs[tiab] OR nomascus[tiab] OR symphalangus[tiab] OR chimpanzee[tiab] OR chimpanzees[tiab] OR prosimians[tiab] OR "bush baby"[tiab] OR prosimian[tiab] OR "bush babies"[tiab] OR galagos[tiab] OR galago[tiab] OR pongidae[tiab] OR gorilla[tiab] OR gorillas[tiab] OR pongo[tiab] OR "pongo pygmaeus"[tiab] OR orangutans[tiab] OR pygmaeus[tiab] OR lemur[tiab] OR lemurs[tiab] OR lemuridae[tiab] OR horse[tiab] OR horses[tiab] OR equus[tiab] OR cow[tiab] OR calf[tiab] OR bull[tiab] OR chicken[tiab] OR chickens[tiab] OR gallus[tiab] OR quail[tiab] OR bird[tiab] OR birds[tiab] OR quails[tiab] OR poultry[tiab] OR poultries[tiab] OR fowl[tiab] OR fowls[tiab] OR reptile[tiab] OR reptilia[tiab] OR reptiles[tiab] OR snakes[tiab] OR snake[tiab] OR lizard[tiab] OR lizards[tiab] OR alligator[tiab] OR alligators[tiab] OR crocodile[tiab] OR crocodiles[tiab] OR turtle[tiab] OR turtles[tiab] OR amphibian[tiab] OR amphibians[tiab] OR amphibia[tiab] OR frog[tiab] OR frogs[tiab] OR bombina[tiab] OR salientia[tiab] OR toad[tiab] OR toads[tiab] OR "epidalea calamita"[tiab] OR salamander[tiab] OR salamanders[tiab] OR eel[tiab] OR eels[tiab] OR fish[tiab] OR fishes[tiab] OR pisces[tiab] OR catfish[tiab] OR catfishes[tiab] OR siluriformes[tiab] OR arius[tiab] OR Page 18 of 20 heteropneustes[tiab] OR sheatfish[tiab] OR perch[tiab] OR perches[tiab] OR percidae[tiab] OR perca[tiab] OR trout[tiab] OR trouts[tiab] OR char[tiab] OR chars[tiab] OR salvelinus[tiab] OR "fathead minnow"[tiab] OR minnow[tiab] OR cyprinidae[tiab] OR carps[tiab] OR carp[tiab] OR zebrafish[tiab] OR zebrafishes[tiab] OR goldfish[tiab] OR goldfishes[tiab] OR guppy[tiab] OR guppies[tiab] OR chub[tiab] OR chubs[tiab] OR tinca[tiab] OR barbels[tiab] OR barbus[tiab] OR pimephales[tiab] OR promelas[tiab] OR "poecilia reticulata"[tiab] OR mullet[tiab] OR mullets[tiab] OR seahorse[tiab] OR seahorses[tiab] OR "mugil curema"[tiab] OR "atlantic cod"[tiab] OR shark[tiab] OR sharks[tiab] OR catshark[tiab] OR anguilla[tiab] OR salmonid[tiab] OR salmonids[tiab] OR whitefish[tiab] OR whitefishes[tiab] OR salmon[tiab] OR salmons[tiab] OR sole[tiab] OR solea[tiab] OR "sea lamprey"[tiab] OR lamprey[tiab] OR lampreys[tiab] OR pumpkinseed[tiab] OR sunfish[tiab] OR sunfishes[tiab] OR tilapia[tiab] OR tilapias[tiab] OR turbot[tiab] OR turbots[tiab] OR flatfish[tiab] OR flatfishes[tiab] OR sciuridae[tiab] OR squirrel[tiab] OR squirrels[tiab] OR chipmunk[tiab] OR chipmunks[tiab] OR suslik[tiab] OR susliks[tiab] OR vole[tiab] OR voles[tiab] OR lemming[tiab] OR lemmings[tiab] OR muskrat[tiab] OR muskrats[tiab] OR lemmus[tiab] OR otter[tiab] OR otters[tiab] OR marten[tiab] OR martens[tiab] OR martes[tiab] OR weasel[tiab] OR badger[tiab] OR badgers[tiab] OR ermine[tiab] OR mink[tiab] OR minks[tiab] OR sable[tiab] OR sables[tiab] OR gulo[tiab] OR gulos[tiab] OR wolverine[tiab] OR wolverines[tiab] OR mustela[tiab] OR llama[tiab] OR llamas[tiab] OR alpaca[tiab] OR alpacas[tiab] OR camelid[tiab] OR camelids[tiab] OR guanaco[tiab] OR guanacos[tiab] OR chiroptera[tiab] OR chiropteras[tiab] OR bat[tiab] OR bats[tiab] OR fox[tiab] OR foxes[tiab] OR iguana[tiab] OR iguanas[tiab] OR "xenopus laevis"[tiab] OR parakeet[tiab] OR parakeets[tiab] OR parrot[tiab] OR parrots[tiab] OR donkey[tiab] OR donkeys[tiab] OR mule[tiab] OR mules[tiab] OR zebra[tiab] OR zebras[tiab] OR shrew[tiab] OR shrews[tiab] OR bison[tiab] OR bisons[tiab] OR buffalo[tiab] OR buffaloes[tiab] OR deer[tiab] OR deers[tiab] OR bear[tiab] OR bears[tiab] OR panda[tiab] OR pandas[tiab] OR "wild hog"[tiab] OR "wild boar"[tiab] OR fitchew[tiab] OR fitch[tiab] OR beaver[tiab] OR beavers[tiab] OR jerboa[tiab] OR jerboas[tiab] OR capybara[tiab] OR capybaras[tiab]) NOT medline[SB])

**Supplementary Table 2**. Key model input parameters.

| **Variable** | **Unit** | **Value** | **Distribution** | **Reference** |
| --- | --- | --- | --- | --- |
| **Demographics of catchment area population** | | | | |
| Women of reproductive age of population in catchment area | % | 19.65 | - | Calculated from the United States Census Bureau (6) |
| Annual fertility rate in the United States | % | 5.45 | - | Calculated from the CDC birth statistics (7) |
| Proportion of GAB25-30 in patient population among births | % | 0.95 | - | Calculated from Wonder database (8) |
| **Length of stay** | | | | |
| Time to NNS training, SoC | Days | 28.3 | Normal | Calculated from Song et al., 2019 (3) |
| Time to NNS training, PFOS | Days | 26.4 | Normal | Calculated from Song et al., 2019 (3) |
| Time to FOF, SoC | Days | 27.0 | Normal | Song et al., 2019 (3) |
| Time to FOF, PFOS | Days | 22.9 | Normal | Song et al., 2019 (3) |
| Time to discharge after FOF achievement | Days | 10.5 | Normal | Calculated from Song et al., 2019 (3) |
| Additional hospital stay before discharge when FOF is not achieved | Days | 5.0 | Normal | Assumption |
| **NNS training effectiveness and time** | | | | |
| NNS training success, SoC | % | 94.06 | Beta | Calculated from Song et al., 2019 (3) |
| NNS training success, PFOS | % | 93.58 | Beta | Calculated from Song et al., 2019 (3) |
| Discharge from NICU to home, non-FOF, SoC | % | 33.33 | Normal | Calculated from Song et al., 2019 (3) |
| Discharge from NICU to home, non- FOF, PFOS | % | 42.86 | Normal | Calculated from Song et al., 2019 (3) |
| Discharge from NICU to home, FOF, SoC | % | 100 | Beta | Calculated from Song et al., 2019 (3) |
| Discharge from NICU to home, FOF, PFOS | % | 100 | Beta | Calculated from Song et al., 2019 (3) |
| Time to discharge factor | - | 0.8 | - | Assumption |
| Training time per day | Minutes | 30 | Normal | Calculated from Cardinal Health Inc, 2025 (9) |
| **Complications** | | | | |
| Infection rates in NICU due to gavage feeding | % | 11.6 | Normal | Lv et al., 2019 (10) |
| Duration of infection | Days | 12 | Normal | Assumption based on Karlowicz et al., 2008 (11) |
| OR rehospitalization due to 1 additional NICU week stay | - | 1.07 | Log-normal | Puthattayil et al., 2021 (12) |
| OR rehospitalization due to gavage feeding at NICU discharge | - | 4.66 | Log-normal | Puthattayil et al., 2021 (12) |
| **Rehospitalization inputs** | | | | |
| Readmission rate during the 30 days post-discharge, 24-27 GAB | % | 30.7 | Normal | Stephens et al., 2016 (4) |
| Readmission rate during the 30 days post-discharge, 28-31 GAB | % | 19.9 | Normal | Stephens et al., 2016 (4) |
| Rehospitalization rate year 1, GAB 24-27 | % | 31.4 | Normal | Calculated from Stephens et al., 2016 (4) |
| Rehospitalization rate year 2, GAB 24-27 | % | 36.0 | Normal | Stephens et al., 2016 (4) |
| Rehospitalization rate year 3, GAB 24-27 | % | 28.4 | Normal | Stephens et al., 2016 (4) |
| Rehospitalization rate year 4, GAB 24-27 | % | 19.3 | Normal | Stephens et al., 2016 (4) |
| Rehospitalization rate year 5, GAB 24-27 | % | 16.8 | Normal | Stephens et al., 2016 (4) |
| Rehospitalization rate year 1, GAB 28-31 | % | 27.8 | Normal | Calculated from Stephens et al., 2016 (4) |
| Rehospitalization rate year 2, GAB 28-31 | % | 25.3 | Normal | Stephens et al., 2016 (4) |
| Rehospitalization rate year 3, GAB 28-31 | % | 18.3 | Normal | Stephens et al., 2016 (4) |
| Rehospitalization rate year 4, GAB 28-31 | % | 15.7 | Normal | Stephens et al., 2016 (4) |
| Rehospitalization rate year 5, GAB 28-31 | % | 13.3 | Normal | Stephens et al., 2016 (4) |
| Proportion of total Rehospitalizations due to infection, year 1 | % | 47.37 | Normal | Ralser et al., 2012 (13) |
| Proportion of total Rehospitalizations due to infection, year 2 | % | 51.95 | Normal | Ralser et al., 2012 (13) |
| Proportion of total Rehospitalizations due to infection, year 3 | % | 40.0 | Normal | Ralser et al., 2014 (14) |
| Proportion of total Rehospitalizations due to infection, year 4 | % | 43.18 | Normal | Ralser et al., 2014 (14) |
| Proportion of total Rehospitalizations due to infection, year 5 | % | 31.25 | Normal | Ralser et al., 2014 (14) |
| **Costs*** | | | | |
| NICU level I cost, per day | USD | 1,285.6 | Gamma | Healthcare Cost Institute (15) |
| NICU level II cost, per day | USD | 2,368.1 | Gamma | Healthcare Cost Institute (15) |
| NICU level III cost, per day | USD | 3,003.9 | Gamma | Healthcare Cost Institute (15) |
| NICU level IV cost, per day | USD | 3,997.8 | Gamma | Healthcare Cost Institute (15) |
| Nurse staff cost (NICU), per hour | USD | 62 | Gamma | Brusie, 2025 (16) |
| Cost of a hospital infection, per case | USD | 1,538.6 | Gamma | Guan et al., 2024 (17) |
| Cost of naso-/orogastric tubes, per case | USD | 3,892.4 | Gamma | White et al., 2020 (18) |
| Cost of Rehospitalization, per case | USD | 12,007.9 | Gamma | Speer et al., 2021 (19) |
| Charge to cost ratio | - | 2.4 | Fixed | Bai and Anderson, 2015 (20) |
| **Acuity levels** | | | | |
| Acuity level before NNS training | - | Level III | - | Assumption |
| Acuity level during NNS training | - | Level III | - | Assumption |
| Acuity level after FOF achievement | - | Level I | - | Assumption |
| Acuity level during additional hospital stay | - | Level III | - | Assumption |
| **Mortality** | | | | |
| All-cause mortality | % | 0.1251 | - | Calculated from the National Center for Health Statistics, ages 0 to 5 (21) |

GAB: gestational age at birth; NNS: non-nutritive sucking; SoC: standard of care; PFOS: patterned frequency-modulated oral stimulation; NICU: neonatal intensive care unit; OR: odds ratio; FOF: full oral feed; NGT: naso-/oro-gastric tube. * Model inputs and results were inflated to and reported in 2024 USD.

**Supplementary Table 3.** Inflation indices

| **Year** | **Consumer Price Index (CPI)** | **Relative CPI** | **Reference** |
| --- | --- | --- | --- |
| 2000 | 266.033 | 2.302045235 | Consumer Price Index for All Urban Consumers: Medical Care Services in U.S. City Average, Index 1982-1984=100, Annual, Seasonally Adjusted  Economic Research Division  Federal Reserve Bank of St. Louis |
| 2001 | 278.758 | 2.19695937 |  |
| 2002 | 292.9 | 2.090884261 |  |
| 2003 | 305.983 | 2.001483743 |  |
| 2004 | 321.35 | 1.905772522 |  |
| 2005 | 336.692 | 1.818932437 |  |
| 2006 | 350.625 | 1.746652406 |  |
| 2007 | 369.318 | 1.658245739 |  |
| 2008 | 384.941 | 1.590945106 |  |
| 2009 | 397.29 | 1.541493619 |  |
| 2010 | 411.198 | 1.489355493 |  |
| 2011 | 423.798 | 1.445075248 |  |
| 2012 | 440.35 | 1.390757352 |  |
| 2013 | 454.011 | 1.348910048 |  |
| 2014 | 464.811 | 1.317567786 |  |
| 2015 | 476.178 | 1.286115696 |  |
| 2016 | 494.77 | 1.237787255 |  |
| 2017 | 506.798 | 1.208410452 |  |
| 2018 | 517.802 | 1.182730078 |  |
| 2019 | 536.127 | 1.142303969 |  |
| 2020 | 562.624 | 1.088506711 |  |
| 2021 | 573.076 | 1.06865407 |  |
| 2022 | 597.704 | 1.024620883 |  |
| 2023 | 595.633 | 1.028183462 |  |
| 2024 | 612.420 | 1 |  |

**Supplementary Table 4.** Economic impact results by FOF achievement for the payer model.

|  | **PFOS** | **Standard of care** | **Difference (95% UI)** |
| --- | --- | --- | --- |
| **FOF group** | | | |
| Pre NNS training NICU | 7,421,225 | 7,996,255 | -575,031 (977,486; -2,110,917) |
| NNS training and follow up | 12,687,514 | 14,409,373 | -1,721,859 (207,373; -3,735,593) |
| **Non-FOF group** | | | |
| Pre NNS training NICU | 509,300 | 505,027 | 4,273 (137,548; -146,802) |
| NNS training and follow up | 1,095,894 | 1,152,587 | -56,693 (198,897; -338,992) |
| **Total costs** | **21,713,932** | **24,063,242** | **-2,349,309 (391,150; -4,958,518)** |

PFOS: patterned frequency-modulated oral stimulation; NICU: neonatal intensive care unit; FOF: full oral feed; NGT: naso-/oro-gastric tube; NNS: non-nutritive sucking. UI: uncertainty interval

**Supplementary Table 5.** NFOF factor variation

| **TTD factor** | **Total difference** | **Variation (from factor = 1)** |
| --- | --- | --- |
| 0.5 | -2,348,728.49 | 0.26% |
| 0.6 | -2,352,116.72 | 0.41% |
| 0.7 | -2,351,680.89 | 0.39% |
| 0.8 | -2,349,309.38 | 0.29% |
| 0.9 | -2,346,072.76 | 0.15% |
| 1.0 | -2,342,562.19 | 0.00% |
| 1.1 | -2,339,090.71 | -0.15% |
| 1.2 | -2,335,812.33 | -0.29% |

NFOF: non-full oral feed; TTD: time to discharge

**Supplementary Figure 1.** Graphical plot of the PFOS cost threshold analysis


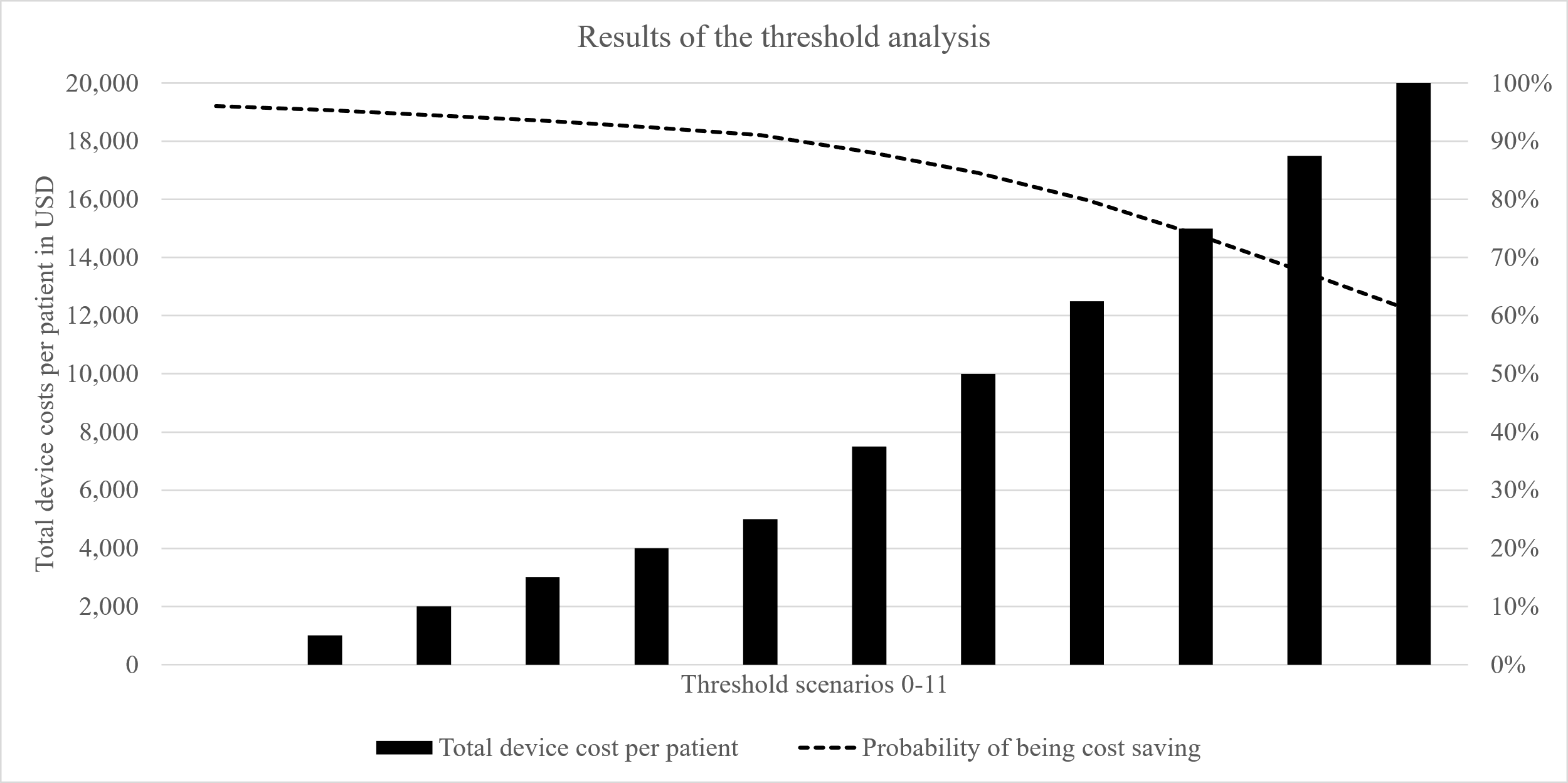


The results of the threshold analysis were computed based on the 5,000-iteration Monte Carlo simulation.

**CHEERS Checklist**

| **Topic** | **No.** | **Item** | **Location where item is reported** |
| --- | --- | --- | --- |
| **Title** |  |  |  |
|  | 1 | Identify the study as an economic evaluation and specify the interventions being compared. | Title |
| **Abstract** |  |  |  |
|  | 2 | Provide a structured summary that highlights context, key methods, results, and alternative analyses. | Abstract |
| **Introduction** |  |  |  |
| **Background and objectives** | 3 | Give the context for the study, the study question, and its practical relevance for decision making in policy or practice. | Page 3 (Background) |
| **Methods** |  |  |  |
| **Health economic analysis plan** | 4 | Indicate whether a health economic analysis plan was developed and where available. | Not available |
| **Study population** | 5 | Describe characteristics of the study population (such as age range, demographics, socioeconomic, or clinical characteristics). | Page 4 (Methods) |
| **Setting and location** | 6 | Provide relevant contextual information that may influence findings. | Page 4 (Methods) |
| **Comparators** | 7 | Describe the interventions or strategies being compared and why chosen. | Page 4 (Methods) |
| Perspective | 8 | State the perspective(s) adopted by the study and why chosen. | Pages 4/5 (Methods) |
| Time horizon | 9 | State the time horizon for the study and why appropriate. | Pages 4/5 (Methods) |
| Discount rate | 10 | Report the discount rate(s) and reason chosen. | Page 3 (Methods) |
| Selection of outcomes | 11 | Describe what outcomes were used as the measure(s) of benefit(s) and harm(s). | Page 3 (Methods) |
| Measurement of outcomes | 12 | Describe how outcomes used to capture benefit(s) and harm(s) were measured. | Not applicable |
| Valuation of outcomes | 13 | Describe the population and methods used to measure and value outcomes. | Not applicable |
| Measurement and valuation of resources and costs | 14 | Describe how costs were valued. | Not applicable |
| Currency, price date, and conversion | 15 | Report the dates of the estimated resource quantities and unit costs, plus the currency and year of conversion. | Page 3 (Methods) |
| Rationale and description of model | 16 | If modelling is used, describe in detail and why used. Report if the model is publicly available and where it can be accessed. | Pages 4/5 (Methods) |
| Analytics and assumptions | 17 | Describe any methods for analysing or statistically transforming data, any extrapolation methods, and approaches for validating any model used. | Page 5/6 (Methods) |
| Characterising heterogeneity | 18 | Describe any methods used for estimating how the results of the study vary for subgroups. | Not applicable |
| Characterising distributional effects | 19 | Describe how impacts are distributed across different individuals or adjustments made to reflect priority populations. | Not applicable |
| Characterising uncertainty | 20 | Describe methods to characterise any sources of uncertainty in the analysis. | Page 5/6 (Methods) |
| Approach to engagement with patients and others affected by the study | 21 | Describe any approaches to engage patients or service recipients, the general public, communities, or stakeholders (such as clinicians or payers) in the design of the study. | Not applicable |
| Results |  |  |  |
| Study parameters | 22 | Report all analytic inputs (such as values, ranges, references) including uncertainty or distributional assumptions. | Supplementary Table 2 |
| Summary of main results | 23 | Report the mean values for the main categories of costs and outcomes of interest and summarise them in the most appropriate overall measure. | Pages 6/7/8 (Results) |
| Effect of uncertainty | 24 | Describe how uncertainty about analytic judgments, inputs, or projections affect findings. Report the effect of choice of discount rate and time horizon, if applicable. | Not applicable |
| Effect of engagement with patients and others affected by the study | 25 | Report on any difference patient/service recipient, general public, community, or stakeholder involvement made to the approach or findings of the study | Not applicable |
| Discussion |  |  |  |
| Study findings, limitations, generalisability, and current knowledge | 26 | Report key findings, limitations, ethical or equity considerations not captured, and how these could affect patients, policy, or practice. | Pages 8/9/10 (Discussion) |
| Other relevant information |  |  |  |
| Source of funding | 27 | Describe how the study was funded and any role of the funder in the identification, design, conduct, and reporting of the analysis | Page 11 (Funding) |
| Conflicts of interest | 28 | Report authors conflicts of interest according to journal or International Committee of Medical Journal Editors requirements. | Page 10 (Conflicts of Interest) |

**Supplementary References**

1. Hooijmans CR, Tillema A, Leenaars M, Ritskes-Hoitinga M. Enhancing search efficiency by means of a search filter for finding all studies on animal experimentation in PubMed. Lab Anim 2010; 44(3):170–5.

2. Sullivan SD, Mauskopf JA, Augustovski F, Jaime Caro J, Lee KM, Minchin M et al. Budget impact analysis-principles of good practice: report of the ISPOR 2012 Budget Impact Analysis Good Practice II Task Force. Value Health 2014; 17(1):5–14.

3. Song D, Jegatheesan P, Nafday S, Ahmad KA, Nedrelow J, Wearden M et al. Patterned frequency-modulated oral stimulation in preterm infants: A multicenter randomized controlled trial. PLoS One 2019; 14(2):e0212675.

4. Stephens AS, Lain SJ, Roberts CL, Bowen JR, Nassar N. Survival, Hospitalization, and Acute-Care Costs of Very and Moderate Preterm Infants in the First 6 Years of Life: A Population-Based Study. J Pediatr 2016; 169:61-8.e3.

5. Silas U, Saunders SJ, Caterino M, Hafermann J, Saunders R, Olatunde A. Procedural management of early pregnancy loss in different hospital settings: a cost-consequence analysis for the USA. J Hosp Manag Health Policy 2024; 8:6.

6. United States Census Bureau. National Population by Characteristics: 2020-2024: <https://census.gov>; 2025 [cited 2025 Apr 15]. Available from: URL: <https://www.census.gov/data/datasets/time-series/demo/popest/2020s-national-detail.html>.

7. Centers for DIseaase Control and Prevention. Natality, 2016-2023; 2025 [cited 2025 Jan 16]. Available from: URL: <https://www.cdc.gov/maternal-infant-health/preterm-birth/?CDC_AAref_Val=https://www.cdc.gov/reproductivehealth/maternalinfanthealth/pretermbirth.htm>.

8. Centers for Disease Control and Prevention. Wonder database: Natality 2013-2023 (Preterm Births): Centers for Disease Control and Prevention; 2023 [cited 2024 Sep 9]. Available from: URL: <https://www.cdc.gov/reproductivehealth/maternalinfanthealth/pretermbirth.htm>.

9. Cardinal Health Inc. Kangaroo NTrainer System 2.0 parent guide | Cardinal Health; 2025 [cited 2025 Jul 3]. Available from: URL: <https://www.cardinalhealth.com/en/product-solutions/medical/enteral-feeding/kangaroo-neonatal-pediatric-feeding-system/ntrainer-system/resources/parent-guide.html>.

10. Lv B, Gao X, Sun J, Li T, Liu Z, Zhu L et al. Family-Centered Care Improves Clinical Outcomes of Very-Low-Birth-Weight Infants: A Quasi-Experimental Study. Front Pediatr 2019; 7:138. Available from: URL: <https://www.frontiersin.org/articles/10.3389/fped.2019.00138/full>.

11. Karlowicz MG, Buescher ES. Nosocomial Infections in the Neonate. Principles and Practice of Pediatric Infectious Disease 2020:543–50. Available from: URL: <https://pmc.ncbi.nlm.nih.gov/articles/PMC7310940/>.

12. Puthattayil ZB, Luu TM, Beltempo M, Cross S, Pillay T, Ballantyne M et al. Risk factors for re-hospitalization following neonatal discharge of extremely preterm infants in Canada. Paediatr Child Health 2021; 26(2):e96-e104.

13. Ralser E, Mueller W, Haberland C, Fink F-M, Gutenberger K-H, Strobl R et al. Rehospitalization in the first 2 years of life in children born preterm. Acta Paediatr 2012; 101(1):e1-5. Available from: URL: <https://pubmed.ncbi.nlm.nih.gov/21767301/>.

14. Ralser E, Elke G, Vera N, Maria G, Michaela H, Ursula K-K. Readmission of Preterm Infants Less Than 32 Weeks Gestation Into Early Childhood: Does Gender Difference Still Play a Role? Glob Pediatr Health 2014; 1:2333794X14549621.

15. Healthcare Cost Institute. NICU Admissions and Spending Increased Slightly from 2017-2021; 2024 [cited 2024 Jul 9]. Available from: URL: <https://healthcostinstitute.org/hcci-originals-dropdown/all-hcci-reports/nicu-use-and-spending-1>.

16. Brusie C. 15 Highest Paying Nursing Jobs In 2025. The Journal of Advanced Practice Nursing 2025 [cited 2025 Jun 5]. Available from: URL: <https://www.asrn.org/journal-advanced-practice-nursing/3249-15-highest-paying-nursing-jobs-in-2025.html>.

17. Guan G, Joshi NS, Frymoyer A, Achepohl GD, Dang R, Taylor NK et al. Resource Utilization and Costs Associated with Approaches to Identify Infants with Early-Onset Sepsis. MDM Policy Pract 2024; 9(1):23814683231226129.

18. White BR, Ermarth A, Thomas D, Arguinchona O, Presson AP, Ling CY. Creation of a Standard Model for Tube Feeding at Neonatal Intensive Care Unit Discharge. JPEN J Parenter Enteral Nutr 2020; 44(3):491–9. Available from: URL: <https://pubmed.ncbi.nlm.nih.gov/31549429/>.

19. Speer RR, Schaefer EW, Aholoukpe M, Leslie DL, Gandhi CK. Trends in Costs of Birth Hospitalization and Readmissions for Late Preterm Infants. Children (Basel) 2021; 8(2). Available from: URL: <https://www.ncbi.nlm.nih.gov/pmc/articles/PMC7916486/>.

20. Bai G, Anderson GF. Extreme Markup: The Fifty US Hospitals With The Highest Charge-To-Cost Ratios. Health affairs (Project Hope) 2015; 34(6):922–8. Available from: URL: <https://pubmed.ncbi.nlm.nih.gov/26056196/>.

21. National Center for Health Statistics, National Vital Statistics System, Mortality, 2020. Available from: URL: https://www.cdc.gov/nchs/nvss/deaths.htm.
